# Rapid Detection and Differentiation of *Legionella pneumophila* and Non*-Legionella pneumophila* Species by Using Recombinase Polymerase Amplification Combined With EuNPs-Based Lateral Flow Immunochromatography

**DOI:** 10.3389/fchem.2021.815189

**Published:** 2022-02-07

**Authors:** Jungang Du, Biao Ma, Jiali Li, Yaping Wang, Tianyu Dou, Shujuan Xu, Mingzhou Zhang

**Affiliations:** Zhejiang Provincial Key Laboratory of Biometrology and Inspection and Quarantine, China Jiliang University, Hangzhou, China

**Keywords:** fluorescent immunochromatographic assay, recombinase polymerase amplification, europium nanoparticles, *Legionella pneumophila*, simultaneous detection

## Abstract

*Legionella*, a waterborne pathogen, is the main cause of Legionnaires’ disease. Therefore, timely and accurate detection and differentiation of *Legionella pneumophila* and non*-Legionella pneumophila* species is crucial. In this study, we develop an easy and rapid recombinase polymerase amplification assay combined with EuNPs-based lateral flow immunochromatography (EuNPs-LFIC-RPA) to specifically distinguish *Legionella pneumophila* and non*-Legionella pneumophila*. We designed primers based on the *mip* gene of *Legionella pneumophila* and the 5S rRNA gene of non-*Legionella pneumophila.* The recombinase polymerase amplification reaction could go to completion in 10 min at 37°C, and the amplification products could be detected within 5 min with EuNPs-LFIC strips. Using a florescent test strip reader, the quantitative results were achieved by reading the colored signal intensities on the strips. The sensitivity was 1.6 × 10^1^ CFU/ml, and a linear standard linear curve plotted from the test strip reader had a correlation coefficient for the determination of *Legionella pneumophila* (*R* ^2^ = 0.9516). Completed concordance for the presence or absence of *Legionella pneumophila* by EuNPs-LFIC-RPA and qPCR was 97.32% (*κ* = 0.79, 95% CI), according to an analysis of practical water samples (*n* = 112). In short, this work shows the feasibility of EuNPs-LFIC-RPA for efficient and rapid monitoring of *Legionella pneumophila* and non-*Legionella pneumophila* in water samples.

## Introduction

*Legionella* is widely found in natural and artificial waters, which can form an aerosol to cause acute infection of the respiratory tract and legionellosis (Albert-Weissenberger et al., 2007). Legionellosis is an infectious disease with a high recessive infection rate and mortality rates, and it presents a great hazard to human health. Patients with the disease usually have fever, chills, and a cough, which may be dry or may produce sputum (Steinert et al., 2007). The first known outbreak of the disease was in Philadelphia, United States, in 1976. A total of 221 people contracted the disease, and 34 died (Fraser et al., 1977). Since the first confirmed cases of *Legionella* in Nanjing in 1982, both epidemic and sporadic cases of the disease have been reported in China (Kang et al., 1982). In 2003, the Chinese inspection and quarantine system developed the industry and national standards for *Legionella* detection and counting based on a conventional culture method in China (HG/T 4323-2012, 2012). Additionally, an international standard method that could detect and count live cells of pathogens by culturing on selective agar plates was developed to test whether water samples met the microbial standards (ISO, 2017). Rapid and accurate determination of the pathogen is the key to the diagnosis and control of the disease.

To date, more than 50 species in the *Legionella* genus have been discovered, and of these, more than 24 species are recognized as agents that cause human illness (Newton et al., 2010). More than 80% of infections are attributed to *Legionella pneumophila* (Lu et al., 2011). Reports also indicate that non-*Legionella pneumophila* are also infectious (Herwaldt et al., 1984). Most infections caused by non-*Legionella pneumophila* arise in immunosuppressed patients (Diederen, 2008). Atypical symptoms from clinical practice make it difficult to distinguish *Legionella pneumonia* from pneumonia arising from other bacterial infections by clinical manifestations alone. Therefore, the identification of *Legionella* species and the differentiation of *Legionella pneumophila* and non-*Legionella pneumophila* species have become increasingly important (Cloud et al., 2000). At present, the reference standard for identifying *Legionella* is still the culture method based on buffered activated carbon and yeast extract medium (BCYE) (Nuthong et al., 2018). In general, about 10 days are required for the entire culture process and biochemical procedures. Compared with traditional culture methods, PCR and other molecular biological detection technology have the advantages of allowing early, rapid diagnosis with high sensitivity and specificity (Yáñez et al., 2005; Boss et al., 2018). However, these approaches are not amenable for wider use in field detection because they require complicated instruments and professional training.

In the past few decades, many nucleic acid isothermal amplification techniques have been developed as valuable on-site diagnostic tools, such as nucleic acid sequence-based amplification (NASBA), rolling circle amplification (RCA), loop-mediated isothermal amplification (LAMP), and recombinase polymerase amplification (RPA) (Compton, 1991; Fire and Xu, 1995; Notomi et al., 2000; Wharam et al., 2001; Vincent et al., 2004; Kurn et al., 2005; Piepenburg et al., 2006). Unliked PCR-based detection methods, the entire reaction process of these isothermal amplification methods is carried out at a constant temperature, which saves the time required for thermal cycling and the cost of precision thermal cycling instruments. Of these methods, the RPA method is the most suitable for field application. First, the working temperature of RPA is lower than LAMP. Portable devices can have a longer battery life because of lower power consumption (Piepenburg et al., 2006; Crannell et al., 2014; Zhao et al., 2015). Second, compared with other isothermal techniques, the RPA method was more tolerant of low concentration samples, and amplification was less affected by inhibition due to impurities (Rosser et al., 2015; Moore and Jaykus, 2017; Ahmed et al., 2018). During the detection phase of the RPA assay, a recombinase is used to facilitate the insertion of oligonucleotide primers into their complementary sequences in double-stranded DNA molecules. Then, using DNA polymerase, a new chain is synthesized from the primer-bound complex, and the entire process is aided by the single-stranded DNA binding protein, which can prevent the dissociation of the primers (Hill-Cawthorne et al., 2014). Similar to PCR, the RPA assay can achieve exponential amplification of the target sequence by using two opposite primers. The amplified products of the RPA assay can typically be detected within 20 min, and the optimal detection temperature is 37°C–40°C (Ma et al., 2017). As a simple, fresh, and fast isothermal amplification technique, RPA demonstrates effectiveness for the detection of a plethora of pathogens, including *Salmonella*, *Vibrio harveyi*, *Listeria monocytogenes*, adenovirus, and microalgae (Santiago-Felipe et al., 2015; Eid and Santiago, 2016; Nybond et al., 2018; Toldrà et al., 2018; Pang et al., 2019).

Two genes are often used in the detection of *Legionella pneumophila* and *Legionella*. The *mip* gene is an important virulence factor, playing an important role in the host cell invasion and intracellular parasitism of *Legionella pneumophila* (Cianciotto et al., 1989). It is reported that the *mip* gene is a specific gene to *Legionella pneumophila* (Jonas et al., 2000). The targets of *Legionella* are mostly the 5S rRNA gene, which is a key gene for distinguishing *Legionella* from other bacteria. Therefore, the *mip* gene and 5S rRNA gene were used as the target fragments.

Many readout devices have been designed to analyze amplification products, but most of them are labor-intensive and expensive, such as electrophoresis systems, sensors, and optical devices for fluorescence detection. For point-of-care testing (POCT), simpler systems such as lateral flow immunochromatography (LFIC) are an option. Traditional lateral flow test strips (LF) use colloidal gold nanoparticles as the labeling material. Despite the detection visualization, the lower detectability of LF provides inadequate analysis performance. To meet POCT sensitivity requirements, a variety of fluorescent particles were applied to LF testing, such as quantum dots (QDs) and time-resolved fluorescent nanobeads (TRFNs) (Wang et al., 2019; Lin et al., 2020; Yu et al., 2020). Compared with the gold nanoparticles, a fluorescence-based detection system has several advantages, such as high sensitivity and objective results. Toxicity limits the widespread application of QDs. The Europium nanoparticle (EuNP) contains thousands of fluorescent chelates in its protective hydrophobic shell, so it has chemical stability and high lanthanide-specific fluorescence, along with extremely high sensitivity and quantitative resolution (Harma et al., 2001; Kokko et al., 2007). Europium nanoparticles are an ideal material for LFIC because of their high fluorescence, long fluorescence lifetime, and low toxicity (Zhou et al., 2012).

In this study, we present an EuNPs-LFIC-RPA assay to detect *Legionella pneumophila* and non-*Legionella pneumophila*. Color signals from the strips are obtained by scanning with the FIC-S2011-B14 fluorescent strip reader (Suzhou Helmen Precise Instruments, Suzhou, Jiangsu, China). Moreover, we have optimized the working concentration and determined the analytical sensitivity and specificity of this approach. Then, we applied this method to identify *Legionella pneumophila* in 112 practical water samples. The results were compared with real-time quantitative PCR (TaqMan probe method). The optimized EuNPs-LFIC-RPA assay reduced the demand for precision instruments, simplified the complex detection process of nucleic acid, and potentially improved to the quantitative diagnosis of *Legionella*.

## Materials and Methods

### DNA Preparation

A total of 24 bacterial strains, including two *Legionella pneumophila* strains, eight non-*Legionella pneumophila* strains, and 14 other bacterial strains (Table 1) were prepared to determine the specificity of the EuNPs-LFIC-RPA assay. Of these, *Legionella pneumophila* (ATCC33152) and non-*Legionella pneumophila* (ATCC 33217) were used as model target strains to optimize the EuNPs-LFIC-RPA assay. Except for the *Legionella* strains, which were inoculated in GVPC liquid medium (Hopebio, Shandong, China), the other strains were all inoculated in Luria-Bertani broth (Sangon, China), and all of them were cultured under similar conditions at 37°C for 48 h. The bacterial cultures were used for the extraction of the genomes or for conventional plate counting. To determine the sensitivity, the *Legionella* strain was grown to the midexponential growth phase and then serially diluted 10-fold in GVPC medium. After that, the bacterial concentration as colony-forming units (CFU) was determined for each dilution by the plate colony counting method.

**TABLE 1:** Information of bacterial strains used for specificity tests in the study.

Strain number	Species	Id of strains	Result of eunps-lfic-rpa	
			*mip* gene	5S rRNA gene
1	*Legionella pneumophila*	ATCC33152	+	+
2	*Legionella pneumophila*	LP-002^a^	+	+
3	*Legionella birminghamiensis*	ATCC 43702	−	+
4	*Legionella wadsworthii*	ATCC 33877	−	+
5	*Legionella bozemanae*	ATCC 33217	−	+
6	*Legionella feeleii*	ATCC 35072	−	+
7	*Legionella adelaidensis*	ATCC 49625	−	+
8	*Legionella gormanii*	ATCC 33342	−	+
9	*Legionella rubrilucens*	ATCC 35304	−	+
10	*Legionella longbeachae*	ATCC 33462	−	+
11	*Salmonella* Enteritidis	GIMCC1.345	−	−
12	*Salmonella* Enteritidis	ATCC 13076	−	−
13	*Vibrio cholera*	GIMCC1.449	−	−
14	*Staphylococcus aureus*	GIMCC1.142	−	−
15	*Staphylococcus epidermidis*	SE-001^a^	−	−
16	*Escherichia coli* O157:H7	GIMCC1.201	−	−
17	*Escherichia coli O157:H7*	ECO-071^a^	−	−
18	*Pseudomonas aeruginosa*	GIMCC1.843	−	−
1^a^	*Shigella sonnei*	GIMCC 1.424	−	−
20	*Shigella flexneri*	CICC 10865	−	−
21	*Vibrio parahaemolyticus*	ATCC17802	−	−
22	*Vibrio parahaemolyticus*	VP-034^a^	−	−
23	*Listeria monocytogenes*	ATCC19115	−	−
24	*Mycobacterium avium*	CMCC 93026	−	−

a Afforded by Zhejiang Academy of Science and Technology for Inspection and Quarantine; +: positive result; ‒: negative result.

GIMCC, Guangdong microbiology culture left, Guangdong, China; ATCC, American type culture collection, Virginia, United States; CICC, China left of industrial culture collection, Shanghai, China; CMCC, national left for medical culture collections, Beijing, China.

We used a lysis buffer method to obtain the DNA of the strains, which made it easier to get DNA from Gram-negative bacteria. The lysis buffer contained 200 mM guanidine hydrochloride, 50 mM Tris-HCl (pH 8.0), 0.01% SDS, and 100 mM NaCl.

DNA extraction from 112 natural water samples were obtained using the boiling method. The concentrated sample was heated at 95°C for 10 min in a portable metal bath (MiniT-100H, Allsheng Instruments Co. Ltd., Hangzhou, China). Afterward, the boiled sample was centrifuged at 6000 rpm in a portable handheld centrifuge (Mini-6KS, Allsheng Instruments Co. Ltd., Hangzhou, China) at room temperature for 5 min. The supernatant could be used as the template for DNA detection.

### Primers and Probes Design

After aligning with Clustal W software, the *mip* gene (GeneBank accession no. AE017354) of *Legionella pneumophila* and 5S rRNA gene (GeneBank accession no. NR_075178.1) of *Legionella* were chosen as the target fragments. Furthermore, according to the analysis in the instruction manual (TwistDx, 2018) and Primer-BLAST (http://www.ncbi.nlm.nih.gov/tools/primer-blast/) combined with Primer 5 and BLAST global alignment, the primers of RPA (Table 2) were carefully designed. For the qPCR method, the pairs of specific primer and probes (Table 2) were designed with Beacon Designer 7.9. Invitrogen Biotechnology Co. Ltd. (Shanghai, China) synthesized all primers and probes.

**TABLE 2:** Sequences of *Legionella* for RPA and qPCR primers/probe.

Primer name	Sequence (5′-3′)	Target gene	Fragment length
RPA primers and probes			
Lep-RPA-F1	5′-GTC​TTA​TAG​CAT​TGG​TGC​CGA​TTT​GGG​G-3′	*Mip*	216 bp
Lep-RPA-R1	5′-Digoxin-CCTTTTACTTTATTTTCATCCGCTTTCTTA-3′		
Lep-RPA-P1	5′-Biotin-TAGCATTGGTGCCGATTTGGGGAAGAATTTT [THF]AAAAATCAAGGCAT-C3-Spacer		
nLep-RPA-F2	5′-GCG​ATT​TGG​AAC​CAC​CTG​ATA​CCA​TCT​CGA-3′	5S rRNA	87 bp
nLep-RPA-R2	5′-Digoxin-CTGGCGATGACCTACTTTCGCATGAGGAAG-3′		
nLep-RPA-P2	5′-FAM-CCTGATACCATCTCGAACTCAGAAGTGAAACATTT [THF]CCGCGCCAATG-C3-Spacer		
qPCR primers and probe			
Lep-rtF1	CAA​GGC​ATA​GAT​GTT​AAT​CC	*Mip*	81 bp
Lep-rtR1	TTCGGTTAAAGCCAATTG		
Lep-rtP1	FAM-CCACTCATAGCGTCTTGCATG-BHQ1		

### Duplex Reaction Protocols for RPA

The target DNA was prepared as described above. Nuclease-free water was used as a negative template control (NTC). In the EuNPs-LFIC-RPA assay, the final reaction system was in a total volume of 50 μl, which contained 2 × reaction buffer (TwistAmp basic kit, TwistDX, Cambridge, United Kingdom), 0.4 μmol/L of each primer, 14 mM Mg-acetate, enzymes, and 2 μl template DNA. Each reaction was first incubated at 37°C for 10 min and then placed on ice. All experiments were performed in triplicate. Afterward, 2 μl RPA amplification product was diluted 50-fold with Tris buffer (pH 8.0), and the diluted product finally added to the lateral flow strips. The sample migrated on the test paper *via* capillary action, and the incubation time of the sample was 5 min.

### Preparation of EuNPs-LFIC

First, the conjugation process was carried out. Two milligrams of carboxylic EuNPs (Shanghai Uni Biotech Ltd., Shanghai, China) was dissolved in 800 μl 2-(N-morpholino) ethanesulfonic acid (MES, 0.05 M, pH 8.2). Then, 30 μl 1-(3-dimethylaminopropyl)-3-ethylcarbodiimide hydrochloride (EDC) was added to the carboxyl EuNPs solution and activated by incubating with slow shaking for 30 min. After activation, excess EDC was removed by centrifugation at 12,000 rpm for 25 min. Then, 1 ml of 10 μg/ml anti-digoxin monoclonal antibody (anti-digoxin mAb) was added to the activated solution. Coupling was performed by gently stirring the mixture at 25°C for 2 h, and the excess antibody was removed by centrifugation at 12,000 rpm for 2 min. Finally, 1 ml of the preservation solution was used to resuspend the precipitate, and the conjugate was stored at 4°C for use. The LFIC contained an absorbent pad, a backing card, a sample pad, and a nitrocellulose (NC) filter membrane. The two test lines of the LFIC were prepared separately with anti-FAM antibody and antibiotin antibody, and the conjugate pad was sprayed with the conjugate of EuNPs and antidigoxin mAb. The immobilized goat antimouse polyclonal antibody (pAb) on the control line was used as the assay control. After the sample pad of EuNPs-LFIC was immersed in phosphate-buffered saline (PBS, 0.01 M, pH7.4) and dried at 37°C for 12 h, it could be stored in a sealed bag at room temperature.

### Method for Comparison

For comparison, the qPCR assay targeting the *mip* gene of *Legionella pneumophila* was used, and primers and probes were as previously described (Table 2). The probe was labeled with FAM fluorophore and BHQ1 quencher. The 20 μl reaction mix contained 10 μl 2 × Premix Ex Taq (Probe qPCR, TaKaRa), 0.2 μl RoxII, 0.8 μM primer sets, 0.1 μM probe, 2.5 μl template DNA, and ddH_2_O. The thermal cycle program was as follows: 95°C for 5 min, 95°C for 10 s, and 60°C for 30 s, the last two steps were repeated for 40 cycles.

### Optimization of EuNPs-LFIC-RPA Conditions

To enhance the efficiency of the EuNPs-LFIC-RPA assay, the reaction time and magnesium ion concentration were optimized. The reaction time optimization was determined by stopping the RPA reaction immediately at eight different times (2.5, 5, 7.5, 10, 12.5, 15, 17.5, and 20 min) after adding magnesium acetate, and then analyzing it immediately on the EuNPs-LFIC. After determining the optimal reaction time for the RPA reaction, the effects of different magnesium ion concentrations on the EuNPs-LFIC-RPA assay, ranged from 0 to 16.8 mM, were studied.

### Sensitivity and Specificity of the EuNPs-LFIC-RPA Assay

In the sensitivity experiment, the extracted DNA of *Legionella pneumophila* and non-*Legionella pneumophila* at concentrations ranging from 10^7^ to 10^0^ CFU/ml were used as the template to add to the reaction. The specificity of the EuNPs-LFIC-RPA assay was assessed with the DNA extracted from 24 bacterial strains. A 3% agarose gel was used to verify whether primer dimer would be produced in RPA reaction, which could lead to false-positive results. All tests were repeated three times.

### Detection of Practical Samples

From 2020 to 2021, a total 112 water samples were collected from air conditioners with sterilized bottles and cooling towers in Zhejiang province in China. Every 200 ml water sample was concentrated using a nitrocellulose filter with a pore size of 0.45 μM (Millipore Company, France). Second, by swirling for 5 min, 30 ml of the water sample to be analyzed was used to resuspend the bacteria collected on the membrane. Then, the boiling method was used to extract the bacterial DNA, which was detected by the EuNPs-LFIC-RPA assay. The EuNPs-LFIC-RPA assay could not only detect the *Legionella genus*, but also differentiate *Legionella pneumophila* and non-*Legionella pneumophila*. The results from qPCR were used as reference. The traditional culture method was conducted according to the International Standard method (ISO, 2017) and Entry&Exit Inspection and Quarantine Industry Standard of the People’s Republic of China (SN/T 2528-2010, 2010).

### Data Analysis

FIC-S2011-B14 fluorescent strip reader software (Suzhou Helmen Precise Instruments, Suzhou, Jiangsu, China) was used to analyze the data collected from EuNPs-LFIC-RPA reaction, and the Bio-Rad CFX Manager and Microsoft Excel software (Microsoft Inc., United States) were used to analyze the data collected from qPCR reactions. The T/C value and the logarithm of the bacterial culture concentration were used to draw the standard curve for EuNPs-LFIC-RPA determination.

In practical samples detection, data of water samples were analyzed by the statistical package SPSS (version 19.0). In SPSS software, Cohen’s kappa coefficient test was used to analyze the concordance for qualitative items. The Mantel Haenszel chi-square test was used to analyze the association between two qualitative variables. A *p* value less than .05 was considered to show a significant difference between the two data sets.

## Results

### Assay Principle

**FIGURE 1:**
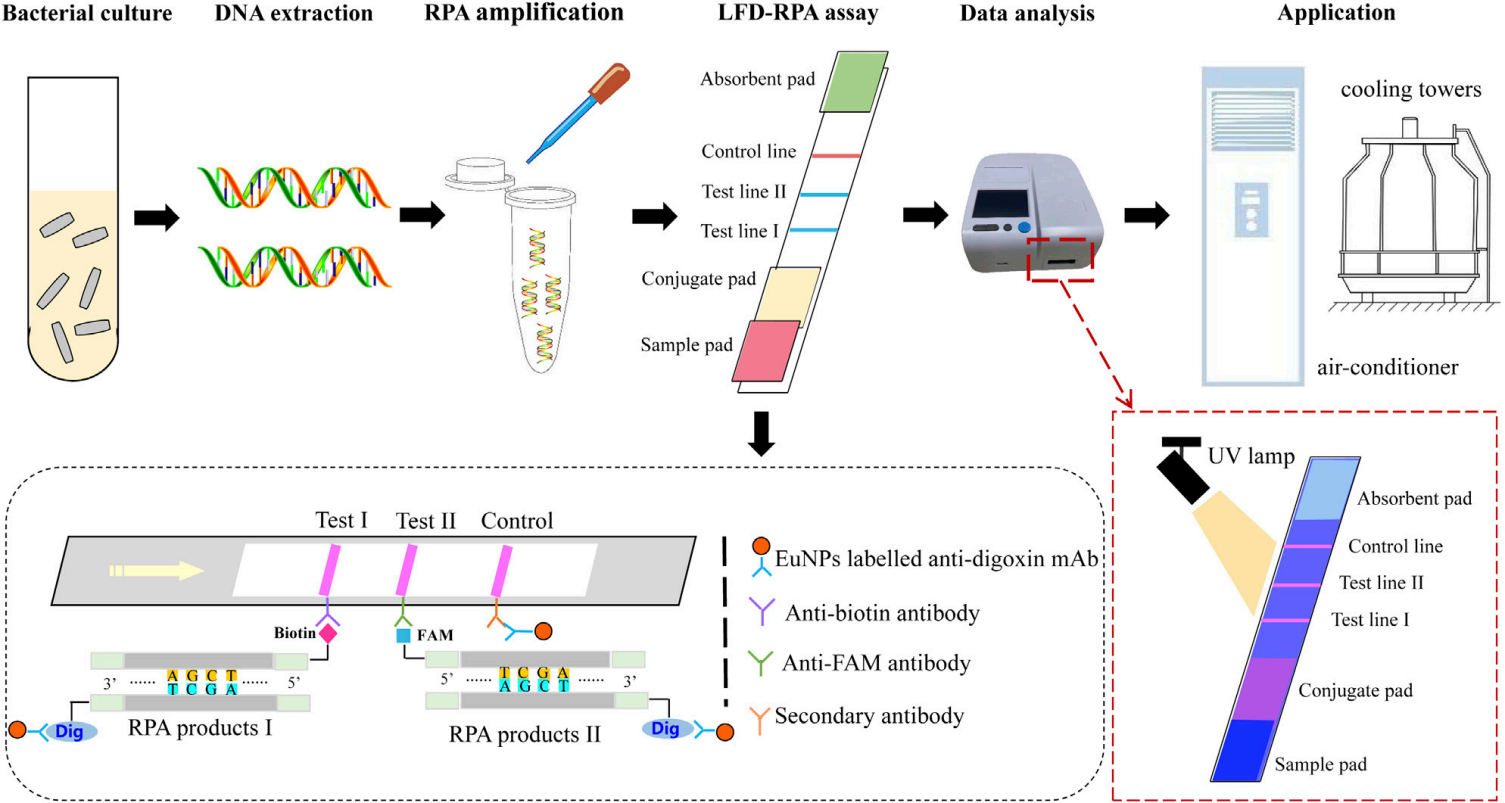
The schematic of EuNPs-LFIC-RPA assay.

The principle of dual EuNPs-LFIC-RPA assay is shown in Figure 1. The RPA reaction was performed to generate amplification products of duplex DNAs, by using a Nfo-probe labeled with carboxyfluorescein (FAM) and biotin, and a downstream primer labeled with digoxin. The sequence of the probe was homologous with the overlapped region of the reverse primer. The Nfo-probe contained a fluorescence label, a C3-spacer, and a tetrahydrofuran (THF), which is an abasic-site mimic and could be identified by the enzyme Nfo (Endonuclease IV). The enzyme Nfo could cleave the probe at the THF position to form a new 3′ hydroxyl substrate, which could continue to extend *via* the polymerase activity of *Bsu* polymerase. Finally, after the RPA amplification products passed through the two detection lines in the EuNPs-LFIC that contained an anti-FAM antibody or an antibiotin antibody, the double-stranded DNA labeled with FAM or biotin would be captured by the corresponding antibody. Goat antimouse polyclonal antibody (pAb) fixed on the control line was used as the analysis control. It could capture the EuNPs, which were not immobilized by the two test lines. In addition, under irradiation with a portable 365 nm UV lamp, the colored result of strips could be observed. The entire procedure took only 5 min. Furthermore, a fluorescent test strip reader was used to scan the strips. This instrument could read the optical signals of the detection line (T) and the control line (C) and convert them into relative electrical signals so as to obtain the ratio of the fluorescence signal intensity of the T and C lines (T/C value).

### Optimization of the EuNPs-LFIC-RPA Conditions

**FIGURE 2:**
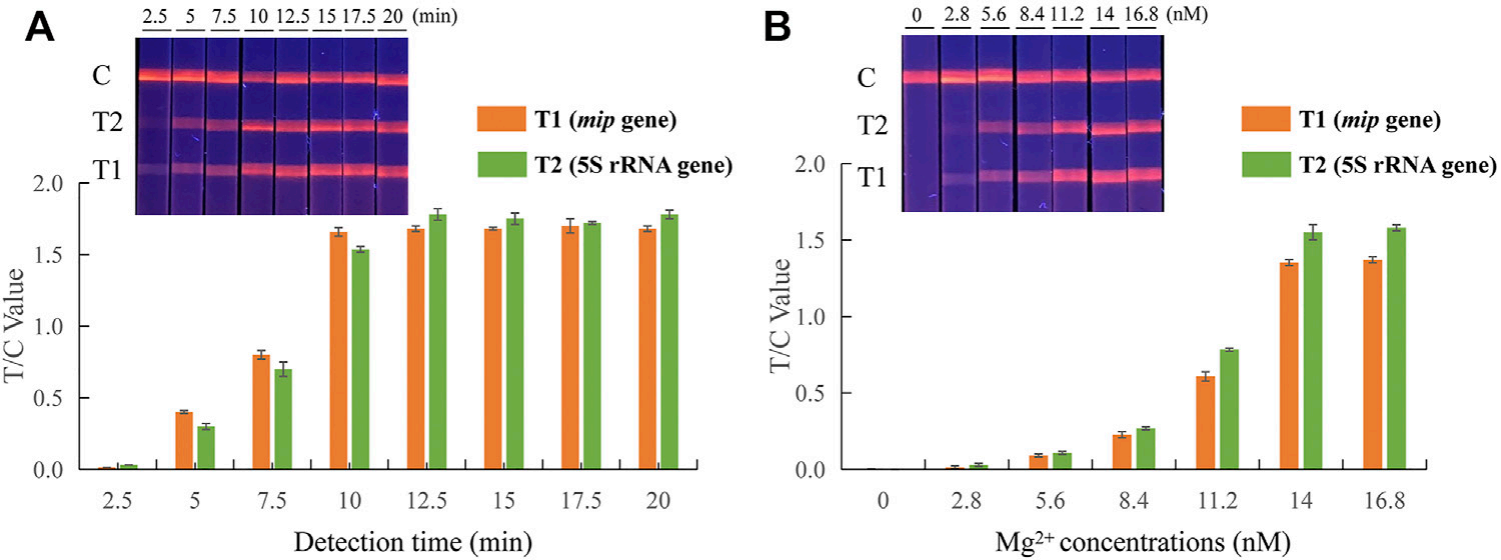
Parameter optimization of dual EuNPs-LFIC-RPA assay. **(A)** incubation time; **(B)** the concentration of magnesium ions.

Several different reaction conditions were tested in the EuNPs-LFIC-RPA assay to obtain the best detection results. The RPA assay was conducted under isothermal conditions at 37°C. Eight time gradients (2.5–20 min) were tested to determine the effect of the incubation time on the amplification efficiency. During the RPA reaction, the longer the amplification time, the greater the brightness of the strips (Figure 2A). At 10 min, the test line had a high fluorescence signal. A subsequent increase of amplification time did not obviously improve the fluorescence signal. Therefore, 10 min was chosen as the appropriate reaction time. Subsequently, six concentrations of 2.8, 5.6, 8.4, 11.2, 14, and 16.8 mM of magnesium ions were tested, and the reaction performed an incubation time of 10 min. The products were analyzed by EuNPs-LFIC. The results showed that, to obtain a higher T/C value while saving reagents, 14 mM of magnesium ion provided the best detection in the EuNPs-LFIC-RPA assay (Figure 2B).

### Sensitivity and Specificity

**FIGURE 3:**
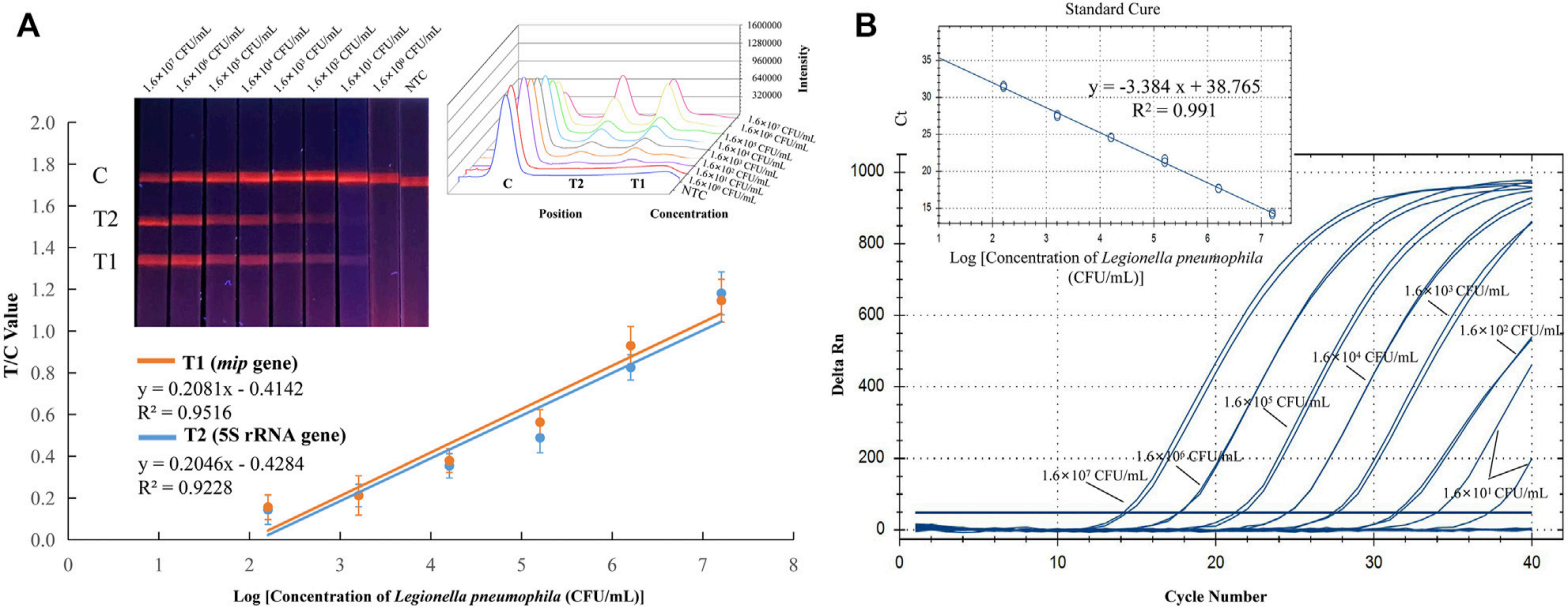
Sensitivity of EuNPs-LFIC-RPA and qPCR assays for *Legionella pneumophila*. Sensitivity was evaluated with *Legionella pneumophila* strain ranging from 1.6 × 10^7^ to 1.6 × 10^0^ CFU/ml. **(A)** The amplified products could be observed with lateral flow strips under 365 nm UV lamp. The intensity was used for quantitative analysis (plotted by Origin 8), and it shows a linear correlation with the concentration of pure cultures. **(B)** The qPCR assay used the same gradient concentration of pure cultures, and the standard curve was generated with the Bio-Rad CFX Manager.

Experiments were carried out to optimize the sensitivity and specificity. The sensitivity was evaluated by using pure cultures of *Legionella pneumophila* and non-*Legionella pneumophila* at concentrations ranging from 10^7^ to 10^0^ CFU/ml. Then, the RPA amplification products were analyzed by EuNPs-LFIC, and the fluorescence signal on the strips was detected using the strip reader. The detection limits of the EuNPs-LFIC-RPA assay for *Legionella pneumophila* and non-*Legionella pneumophila* was 1.6 × 10^1^ CFU/ml and 2.2 × 10^1^ CFU/ml, respectively (Figure 3A; Supplementary Figure S1). The detection limit of the qPCR method for the detection of *Legionella pneumophila* was 1.6 × 10^2^ CFU/ml (Figure 3B). The standard curve for *Legionella pneumophila* (*R* ^2^ = 0.9516) determined by EuNPs-LFIC-RPA shows a significant correlation between the detection threshold and the template concentration. The standard curve for the qPCR assay had a correlation coefficient of determination of 0.991 for *Legionella pneumophila* (Figure 3B).

**FIGURE 4:**
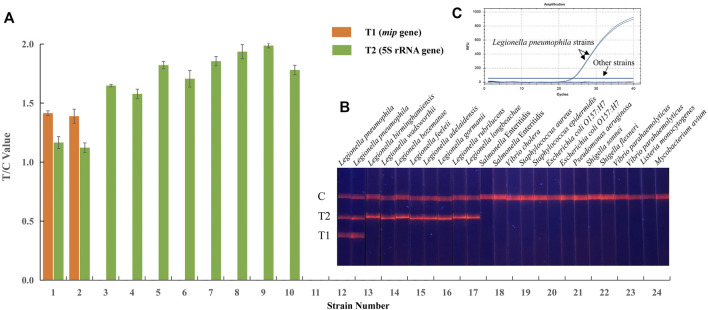
Specificity of EuNPs-LFIC-RPA and qPCR assays for *Legionella pneumophila*. **(A,B)** The light signal was read out by the FIC-S2011-B14 fluorescent strip reader. **(C)** The similar results obtained by qPCR assays.

To determine the specificity, a total of 24 reference strains were investigated in EuNPs-LFIC-RPA experiments, including two *Legionella pneumophila* strains, eight non-*Legionella pneumophila* strains, and 14 other bacterial strains. Genomic DNA extracted from these strains was used as templates for the detection in both EuNPs-LFIC-RPA and the qPCR method. The results show that only the *Legionella pneumophila* strain and non-*Legionella pneumophila* showed positive results (Figures 4A,B). There was no cross-reactivity among *Legionella* strains. Furthermore, the qPCR test results were consistent with the EuNPs-LFIC-RPA (Figure 4C).

Moreover, a primer dimer might be produced during the process of amplification, which could cause false positive results and interfere with the evaluation of the detection results. Therefore, agarose gel electrophoresis was used to analyze the amplification products of RPA testing (Supplementary Figure S2). The results show that two different bands were clearly displayed in the dual RPA reaction, whereas there was no band in the negative control. Therefore, the effect of a primer dimer was neglected.

### Detection of Practical Water Samples

EuNPs-LFIC-RPA and qPCR detection were used on 112 collected water samples. The EuNPs-LFIC-RPA test results indicated eight positive water samples, meaning that the water samples were contaminated with *Legionella pneumophila*. A total of seven water samples were positive in the qPCR test. Therefore, the concordance of EuNPs-LFIC-RPA and the qPCR assay for the detection of *Legionella pneumophila* was 97.32% (Table 3), which demonstrated high consistency between the two methods.

**TABLE 3:** Concordance between EuNPs-LFIC-RPA and qPCR tests in specimens for detection of *Legionella pneumophila*.

Test result	Numbers of samples (%) with qPCR		Total numbers of samples (%)	Absolute agreement (%)	Kappa value (95% CI)
	Lep (+)	Lep (−)			
LF-RPA result					
Lep (+)	6	2	8 (7.14)	97.32	0.79
Lep (−)	1	103	104 (92.86)		
Total	7 (6.25)	105 (93.75)	112		

CI, confidence interval; Lep (+) result refers to *Legionella pneumophila* detection, Lep (−) result reflects the absence of *Legionella pneumophila*.

## Discussion

*Legionella pneumophila* is the most common and severe conditional pathogen in the genus *Legionella*. However, other non-*Legionella pneumophila* are also infectious. They can all cause respiratory disease in humans. Therefore, the rapid detection of *Legionella* is of great significance for the protection of human health. By using a thermal sensor instead of the visual detection of growth, the culture-based isothermal microcalorimetry (IMC) method could shorten the detection time to 24–48 h (Fricke et al., 2020). As the gold standard, the qPCR assay appears useful for applications in detection of *Legionella* bacteria (Kontchou and Nocker, 2018; Toplitsch et al., 2018). Furthermore, ddPCR has also been studied for the detection of *Legionella pneumophila*, and it was more accurate and robust than qPCR (Falzone et al., 2020). However, these PCR-based detection methods all require expensive thermal cycling equipment to perform multiple cycles between two and three temperatures, so they are generally used for laboratory testing instead of on-site testing. For convenience and simplicity, a real time loop-mediated isothermal amplification platform was developed to detect *Legionella* bacteria in tap water in 2 h (Samhan et al., 2017). This detection method is highly sensitive, but it is also more susceptible to aerosol pollution and amplification inhibition. In contrast, the recombinase polymerase amplification assay is more resistant to inhibitors and can also quickly achieve high-sensitivity nucleic acid detection outside the laboratory (Li et al., 2018). For the detection of *Legionella pneumophila*, there have been a few reports of its application with RPA (Kersting et al., 2018; Kober et al., 2018). Combined with lateral flow test strips, the RPA method could detect *Legionella pneumophila* within 20 min (Kersting et al., 2018). In this study, we have developed the EuNPs-LFIC-RPA method for the detection of *Legionella pneumophila* and non-*Legionella pneumophila.* The EuNPs have unique photoluminescent properties, including a clear emission profile, a large stokes shift, a long emission fluorescence lifetime, and a high emission fluorescence intensity (Luo et al., 2017). To ensure efficiency, the EuNPs-LFIC-RPA technology could detect *Legionella pneumophila* at concentrations as low as 1.6 × 10^1^ CFU/ml with a detection time of less than 15 min. In addition, we replaced the DNA extraction kit with the lysis buffer method, which made it easier to obtain bacterial DNA and more compatible with application at the point of care. We estimated a reagent cost for qPCR of about $20 per assay. However, the cost per test is less than $10 for the EuNPs-LFIC-RPA assay described in this paper.

Several attempts were made to improve the detection performance of the EuNPs-LFIC-RPA assay. For the RPA reaction, the amplification time is an important factor affecting detection. The starting time of the reaction can be controlled by the addition of magnesium ions. Once the magnesium ions enter the system, the reaction starts immediately. After a series of experiments, an amplification time of 10 min proved to have the greatest effect on detection for the RPA reaction. In addition, the concentration of magnesium ions also affects the efficiency of amplification. The signal intensity of the test point observed when the concentrations of the magnesium ions exceeded 5.6 mM. However, the optimal concentration should balance the reaction rate and the quantitative accuracy and 14 mM was finally considered as the optimal concentration of magnesium ions in the EuNPs-LFIC-RPA assay. The optimized EuNPs-LFIC-RPA reaction could go to completion in 15 min. At a high template concentration, the detection time could be further reduced. The entire EuNPs-LFIC-RPA assay can be considered in two steps. First, the RPA amplification reaction amplifies the target DNA, and then the amplified product is diluted and used for the visual detection on the EuNPs-LFIC strips.

Compared with other detection methods, the EuNPs-LFIC-RPA assay has a shorter detection time and is easier and simpler to use (Supplementary Table S1). The reaction temperature is another very important contributing extrinsic factor. A water bath or heating block could be used to carry out the EuNPs-LFIC-RPA assay. Even when the weather is hot, such as the summer temperature in continental areas, the EuNPs-LFIC-RPA assay could be carried out at room temperature (Lillis et al., 2014). Compared with other isothermal amplification assays, in which the operating temperature was higher than 60°C, the EuNPs-LFIC-RPA assay can reduce power consumption for diagnostic tests by using a comparatively lower temperature.

In addition, a portable florescent test strip reader could be applied to scan the test strips and analyze the intensity of colored signals. A photodiode combined with modern optoelectronic technology is employed by the test strip reader to detect the intensity of the reflected light. Such a test strip reader could avoid errors caused by reading with the naked eye and improve the sensitivity and accuracy of detection. The EuNPs-LFIC-RPA technology with the test strip reader could not only achieve rapid and accurate detection, but also has the potential for quantitative detection in the field. The challenges for high-throughput and differentiating viable cells from dead cells will be the focus of future research.

## Conclusion

The EuNPs-LFIC-RPA assay described in this paper can not only perform high-sensitivity and strong-specific detection in a short time, but is also very easy and simple to implement. With a portable fluorescent strip reader, it can be used as a rapid quantitative detection assay without a complex thermal cycling machine. Moreover, the application can distinguish *Legionella pneumophila* and non-*Legionella pneumophila*, and no cross-reactions have been observed. In summary, the EuNPs-LFIC-RPA is a practical, simple-to-conduct method for which no or little instrumentation is necessary. This fast and easy read-out system could be useful as a rapid detection method to *Legionella pneumophila* in the field.

## Data Availability

The original contributions presented in the study are included in the article/Supplementary Materials, further inquiries can be directed to the corresponding author.
