# Supplementary material for: Rapid Detection and Differentiation of Legionella pneumophila and Non-Legionella pneumophila Species by Using Recombinase Polymerase Amplification Combined With EuNPs-Based Lateral Flow Immunochromatography
Source: Front Chem. 2022 Feb 7;9:815189. doi: 10.3389/fchem.2021.815189 (PMC8859533; doi:10.3389/fchem.2021.815189)
Supplement: Supplementary file 1 [file DataSheet1.docx]

**Supplementary material**

**
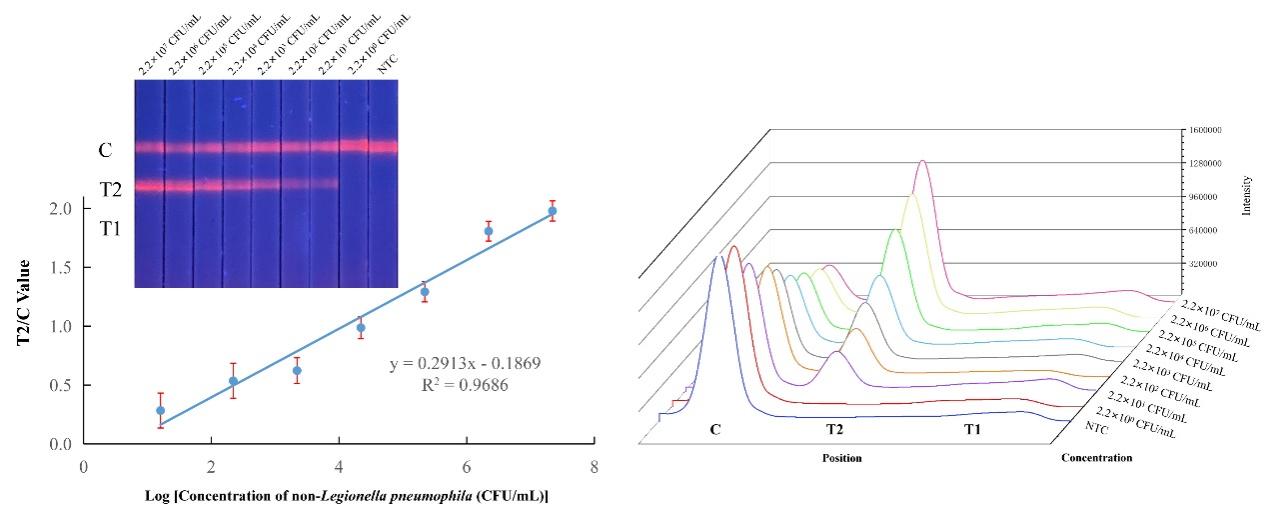
**

**Fig. S1 Sensitivity of EuNPs-LFIC-RPA assays for non-*Legionella pneumophila*.**

The concentration of non-*Legionella pneumophila* strain was ranging from 2.2×10^7^ to 2.2 ×10^0^ CFU/mL. The amplified products could be observed with lateral flow strips under 365 nm UV lamp. The intensity was used for quantitative analysis (plotted by Origin 8), and it shows a linear correlation with the concentration of pure cultures.

**
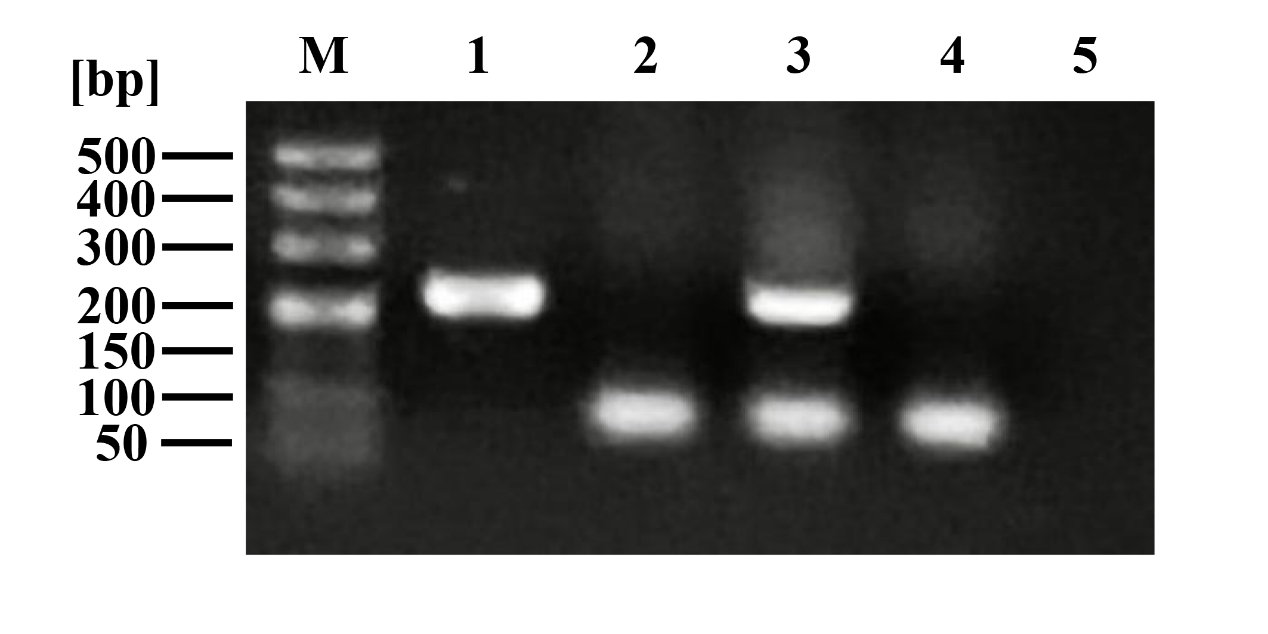
**

**Fig. S2 Agarose gel electrophoresis results of RPA amplification products of *Legionella pneumophila* and *non-Legionella pneumophila*.**

1-2: single RPA; 3-4: dual RPA. The fragment size is 216 bp for *mip* gene of *Legionella pneumophila*. The fragment size is 87 bp for 5s rRNA gene of non-*Legionella pneumophila*. M: marker; 1: Lep-RPA primers *+ L. pneumophila*; 2: nLep-RPA primers + non-*Legionella pneumophila*; 3: Lep-RPA primers *+* nLep-RPA primers + *L. pneumophila*; 4: Lep-RPA primers *+* nLep-RPA primers + non-*L. pneumophila*; 5: no template control.

**Table S1. Comparison of the proposed method for *Legionella pneumophila* detection with other methods.**

| Method | Analyte | LOD | Detection time | Reference |
| --- | --- | --- | --- | --- |
| IMC | L. *pneumophila* | 100 CFU/mL | 24-48 hours | Fricke, C., et al. 2020 |
| LF-RPA | L. *pneumophila* | 10 CFU/reaction | <20 minutes | Kersting, S., et al.2018 |
| ddPCR | L. pneumophila | 10^0^ CFU/mL | >1 hour | Falzone, L, et al. 2020 |
| qPCR | L. *pneumophila* | 2×10^2^ GU/100 mL | >1 hour | Toplitsch, D., et al. 2018 |
| Culture method | L. *pneumophila* | 10 CFU/L | 7-10 days |  |
| EuNPs-LFIC-RPA | L. *pneumophila* | 1.6×10^1^ CFU/mL | <15 minutes | This study |

IMC: isothermal microcalorimetry;

LF-RPA: lateral flow recombinase polymerase amplification;

GU: Genome Units
